# An anomalous interlayer exciton in MoS_2_

**DOI:** 10.1038/srep37075

**Published:** 2016-11-14

**Authors:** Dilna Azhikodan, Tashi Nautiyal, Sam Shallcross, Sangeeta Sharma

**Affiliations:** 1 Department of Physics, Indian Institute of Technology Roorkee, Uttarakhand - 247 667, India; 2 Lehrstuhl für Theoretische Festkorperphysik, Staudtstr. 7-B2 91058 Erlangen, Germany; 3 Max Planck Institute of Microstructure Physics, Weinberg 2, D-06120 Halle, Germany

## Abstract

The few layer transition metal dichalcogenides are two dimensional materials that have an intrinsic gap of the order of ≈2 eV. The reduced screening in two dimensions implies a rich excitonic physics and, as a consequence, many potential applications in the field of opto-electronics. Here we report that a layer perpendicular electric field, by which the gap size in these materials can be efficiently controlled, generates an anomalous inter-layer exciton whose binding energy is independent of the gap size. We show this originates from the rich gap control and screening physics of TMDCs in a bilayer geometry: gating the bilayer acts on one hand to increase intra-layer screening by reducing the gap and, on the other hand, to decrease the inter-layer screening by field induced charge depletion. This constancy of binding energy is both a striking exception to the universal reduction in binding energy with gap size that all materials are believed to follow, as well as evidence of a degree of control over inter-layer excitons not found in their well studied intra-layer counterparts.

The experimental fabrication of graphene in 2004 marked the onset of a new science of low dimensional materials. Of the many materials that have followed graphene, one of the most remarkable has been the few layer transition metal dichalcogenides^1^,^2^,^3^ (TMDCs). Similar to graphene in some ways, for instance both have a valley degree of freedom^4^, these materials differ in a number of key respects. While graphene is a semi-metal the TMDCs have an intrinsic band gap, an inheritance from the bulk crystal which is a semi-conductor. Furthermore, a high degree of control over this gap exists in bilayer TMDCs^5^, with theory^6^,^7^ reporting that layer perpendicular electric fields of only 2–3 V/nm can close a gap of ≈2 eV. As a band gap plays a crucial role in the on-off ratio of field-effect transistors, the presence of an intrinsic and controllable band gap is a profound technological advantage^8^,^9^. Most importantly, the TMDCs host a rich physics of excitons (a bound state formed of a valence band hole and a conduction band electron) with exceptionally large binding energy^5^,^10^,^11^,^12^,^13^,^14^,^15^,^16^. This opens up a vast arena of potential opto-electronic applications for these materials^17^. In this paper, through the example of MoS_2_, we will investigate the theory of the interplay between these two key aspects of TMDCs: the electric field control of the band gap and the excitonic physics.

Theoretical understanding of the TMDCs is non-trivial. Firstly, there are many indications that electron correlation plays a decisive role in the properties of these materials^6^,^18^, a point of view we will richly confirm in this paper. Secondly, excitonic physics by its very nature demands a many-body treatment. A standard density functional theory approach will not suffice for the investigation of these materials and recourse must be made to the full machinery of many-body physics: the *GW* method must be deployed for the ground state followed by the solution of the Bethe-Salpeter equation^19^ (BSE), or a time dependent density functional theory^20^, to access the excitonic physics. These many-body techniques are computationally very demanding and thus only a few such calculations^6^,^21^,^22^ exist, all of which are for the case of zero electric field.

A recent experimental investigation reports an intriguing excitonic physics of MoS_2_ in the presence on a layer perpendicular electric field, with intra- and inter-layer excitons appearing to behave quite differently^5^. It is the purpose of the present paper to apply the full “tool kit” of many-body *ab*-*initio* science (*GW* in conjunction with the BSE) to this system, and to fully reveal the remarkable nature of this excitonic physics of MoS_2_ in an electric field.

## Results and Discussion

### Band gap engineering

**Figure 1:**
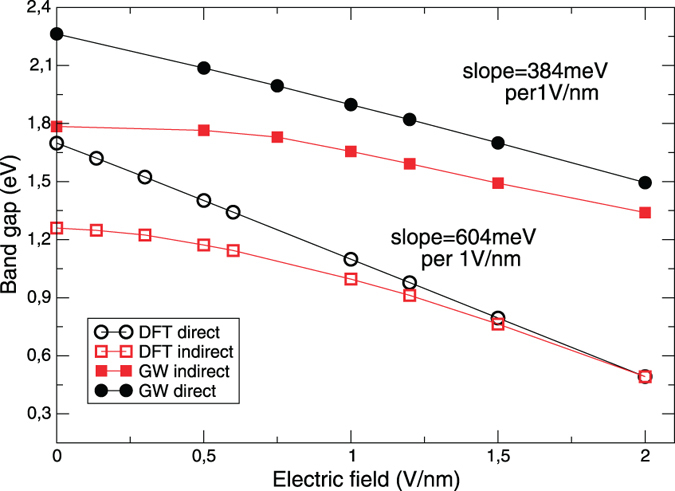
Direct and indirect band gaps (in eV) as a function of electric field (in V/nm). Calculations are performed using *GW* method and DFT.

The behaviour of bilayer MoS_2_ in a layer perpendicular electric field has been well established by DFT studies: the band gap can be continuously reduced with increasing field strength^5^,^23^,^24^,^25^. This tuning of the band gap is so dramatic that both (i) a transition from indirect to direct band gap and (ii) full metalization can be observed (at 1–2 V/nm and 2–3 V/nm respectively, depending on the stacking type of the bilayer). As the ground state spectrum is known to be strongly - and non-uniformly - renormalized by many-body effects, we will first revisit the band gap behaviour using the *GW* method. In Fig. 1 we present both DFT (we deploy the PBE functional) and *GW* calculations of the band gap as a function of field; we show results only for the *AA*′ stacking, known to be the lowest energy stacking configuration^26^ of the MoS_2_ bilayer. Clearly, many-body effects play a crucial role in the behaviour of the band gap: while the gap reduces at 604 meV per unit field with the DFT calculation (in good agreement with earlier work^23^,^25^, this value is almost half −384 meV - if many-body effects are included via the *GW* method. These *GW* results are much closer to the experimental value^5^ of 260 meV per unit field. We also note that the band gap error from the DFT calculation appears to be increasing with field: an error of 30% at zero field becomes an error of almost 70% at 2 V/nm. This strongly suggests that many-body effects are both crucial to the treatment of bilayer MoS_2_ in an electric field, and indeed become more important with increasing field. As the difference between the two methods lies in the much better treatment of screening in the *GW* method, and as excitonic physics depends crucially on the screening of the excitons, we conclude that a *GW* treatment of the ground state is essential.

### Field induced excitonic physics

**Figure 2:**
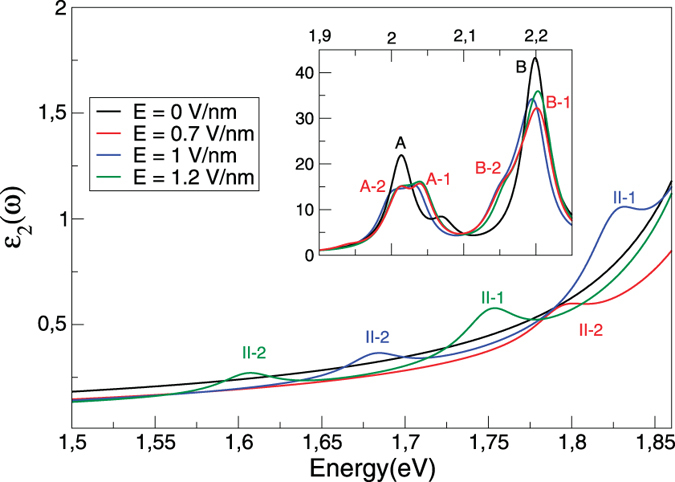
The imaginary part of the in-plane dielectric function of bilayer MoS_2_. Results are presented for four different values of the layer perpendicular field as indicated. The inset presents the behaviour of *ε*_2_ over a wide energy range. Two distinct types of excitonic behaviour may be observed: an exciton (A) whose resonance energy does not change as a function of the electric field, and a second exciton (II) whose resonance energy changes strongly with applied field. All results are calculated by solving BSE in presence of an electric field and spin-orbit coupling.

The imaginary part of the dielectric function *ε*_2_, calculated by solving the BSE, is exhibited in Fig. 2 for a range of electric fields. Below the fundamental gap and in absence of the electric field *ε*_2_ is dominated by two main excitonic peaks, marked as A and B at energies 1.99 eV and 2.19 eV respectively (these peak positions are in excellent agreement with previous theoretical works^18^,^21^). When the field is applied two things occur: (a) the A and B peaks split, but otherwise remain largely unchanged in energy as the field changes and (b) above a critical field of 0.3 V/nm a new spin - orbit split excitonic peak (II-1 and II-2) emerges whose resonance energy changes strongly as a function of field. The second of these features is the novel interlayer excitonic peak described in the experimental work of Chu *et al.* (the spin - orbit splitting is likely too small to have been observed, see the broad peaks in Fig. 4c and error bars in Fig. 4e of ref. 5). While the experiment clearly observed a dramatically different behaviour of peak positions A and II, measurement accuracy precluded a statement being made about the binding energies (difference between the gap and the position of the excitonic peak) of these excitons. Theoretically we have no such restriction, and can now proceed to a careful analysis of this novel excitonic behaviour.

We first confirm the intra- and inter-layer character of the exciton types A and II. This may be easily checked as it is well established that all bands near the gap approach pure layer character as the field increases. Thus by restricting the sub-space in which the Bethe-Salpeter equation is solved to conduction and valence manifolds from the same, or different, layers we may immediately deduce which excitons result from intra- or inter-layer transitions. We are thus, in this way, able to confirm that exciton peak A (and B) are purely intra-layer excitons, while exciton peak II is an inter-layer exciton.

**Figure 3:**
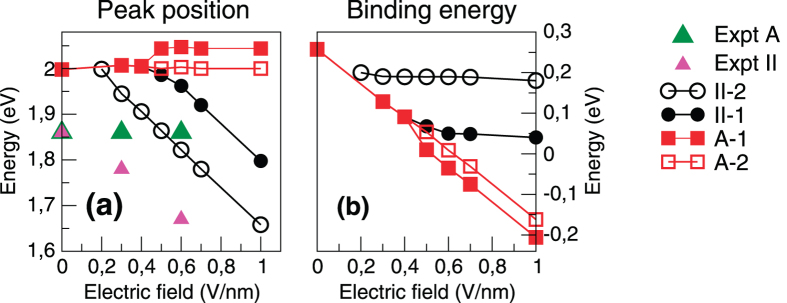
(**a**) Excitonic resonance energy and (**b**) binding energy (difference between the position of the excitonic peak and the *GW* direct band gap) as a function of the electric field applied to a MoS_2_ bilayer. Experimental data for the peak position from ref. 5 are also presented for comparison. Note the opposite trend of peak position and binding energies for the excitons: while exciton II has a peak position that depends strongly on applied field its binding energy is constant, and vice versa for exciton A.

Having established this, in Fig. 3(a) we first present the peak position as a function of applied field, finding agreement with experiment in the fact that the A-1 and A-2 peaks remain almost unchanged with field while, on the other hand, the position of the II-1 and II-2 peaks changes strongly. The absolute positions of all resonances are blue shifted with respect to experiments^5^,^10^ by ~0.14 eV, a fact that has already been noticed at zero field^21^. This deviation from experiment is almost independent of the applied field, and we therefore find a rate of change of peak II with applied field to be very close to the experimental value of 275 meV per unit field (the best find line to the theoretical data yields 384 meV per unit field). We now turn to the binding energy of excitons A and II, presented in Fig. 3(b). Remarkably, the evolution in the position of peak II with field is almost perfectly compensated by a corresponding change in the band gap. On the other hand the constant peak position of exciton A-2, due to the changing band gap, corresponds to a continuously reducing binding energy with field (exciton A is not bound for field values ≥0.6 *V*/*nm*). We therefore conclude that the trends in binding energy - the physically relevant property of the exciton - are the opposite of the peak position trends: the binding energy of exciton II is *weakly dependent* on the field, while that of A is *strongly dependent on* the field.

**Figure 4:**
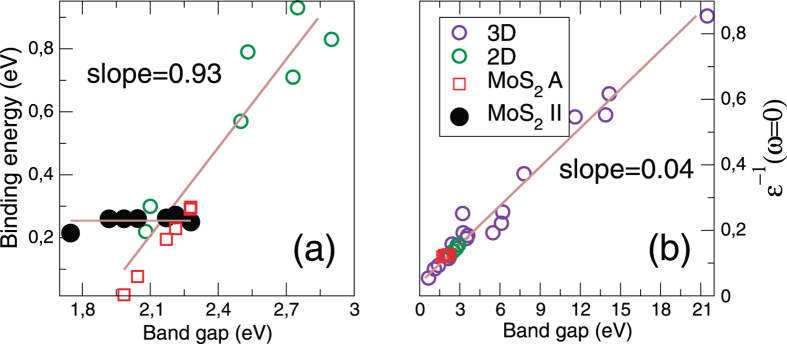
(**a**) Exciton binding energy and (**b**) static screening as a function of the band gap. In panel (**a**) we see that the binding energy of the inter-layer exciton (black circles) is almost independent of the gap size, dramatically violating the universal law that all other materials follow. The two lines are best fits to the inter-layer exciton and all other materials. In panel (**b**) we display static screening a diverse set of two and three dimensional materials have been studied. 3D materials in the order of increasing band gaps: Ge, Si, GaAs, AlAs, ZnTe, SiC, ZnSe, SrTiO_3_, BaTiO_3_, ZnS, Diamond, BN, AlN, NaH, Kr, LiF, Ar, Ne. 2D materials: mono- and bi- layers of MoS_2_, MoSe_2_, WS_2_ and WSe_2_ in zero field. Evidently, there is a very well followed universal linear law for the dependence of static screening on gap size.

An exciton binding energy that is almost independent of the gap is a strikingly anomalous behaviour, a point that may be vividly brought out by comparison to the gap dependence of binding energies found generally in two dimensional materials. In Fig. 4(a) we present the binding energy as a function of gap size for a wide range of two dimensional materials. In the absence of the anomalous exciton II, all binding energies fall close to a best fit line, a universal behaviour first noticed by Choi *et al.*^27^. In Fig. 4(a) we include several additional materials to those considered in ref. 27 which both changes the slope of the universal line and worsens the fit, however to a good approximation the linear law still holds. What lies behind this universal relation discovered by Choi *et al.* To answer this in panel (b) of Fig. 4 we present macroscopic *ε*^−1^ (*ω* → 0, **q** = **0**) - a direct measure of the screening - plotted against the gap size for a diverse set of materials (including both two and three dimensional cases). An almost perfect universal linear behaviour can be observed, and thus the linear law for the exciton binding energy^27^ represents a manifestation of a deeper linear screening law. The constancy of the binding energy of exciton II as a function of the band gap in bilayer MoS_2_, in conjunction with the “regular” behaviour of exciton A, is therefore indicative of an unusual screening physics.

To further elucidate this physics we note that in an increasing electric field the charge accumulates in-plane while being depleted from the interlayer region^23^. As a consequence of this field induced change in charge density intra-layer screening will become stronger with increasing field (more charge to screen with) while, at the same time, having a tenancy to reduce inter-layer screening (less charge to screen with). This explains the binding energy trend of both excitons: the in-plane exciton (A) is “screened away” due to this increased intra-layer screening while, in contradistinction, once the interlayer screening is sufficiently weak the binding energy of exciton II is insensitive to further reduction in screening. This critical value occurs at a field strength of ≤0.3 *V*/*nm* and below this value distinct II and A excitons cannot be determined. By performing a BSE calculation in the subspace of only inter-layer bands we confirm that the exciton II is created at a field of 0.18 V/nm.

**Figure 5:**
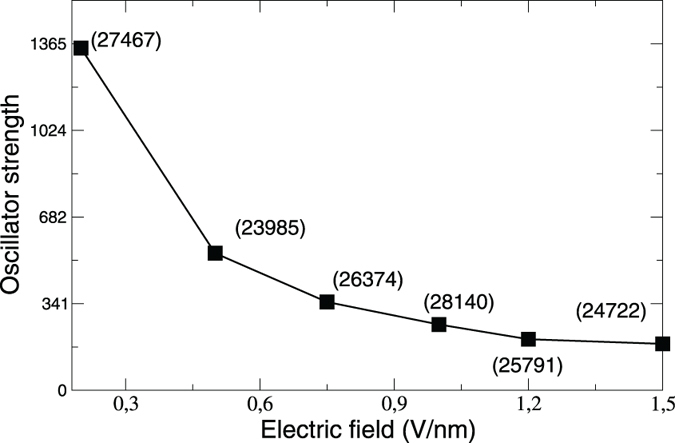
Oscillator strength, calculated by solving the BSE, as a function of electric field for peak II. At each point the oscillator strength for peak A-1 is written in brackets.

It is important to mention that the new inter-layer exciton II is bright but, as is already clear from Fig. 2, comes with a very low intensity. To probe this further we plot in Fig. 5 the oscillator strength (OS)^19^ for peak II as a function of field. Evidently, the exciton II OS is not only very low, for instance at 0.1 V/nm the OS for peak A is about 20 times greater, but furthermore continuously reduces with increasing field strength; at 1.5 V/nm this ratio of oscillator strengths is 140. These results are in perfect consonance with the experimental finding of a very low photoluminescence yield for the inter-layer exciton as compared to the intra-layer exciton.

## Summary and Discussion

Gating the bilayer creates a new inter-layer charge transfer exciton, recently reported in experiments^5^. We find excellent agreement with experimental measurements of the exciton peak positions, and in addition our theoretical analysis affords considerable additional insight into the nature of the excitonic physics. In particular, we find that the interlayer exciton (II) has a binding energy that is, quite remarkably, independent of the gap size. We trace this anomalous behaviour to the fundamental screening physics of the bilayer: an electric field acts to increase intra-layer screening but decrease inter-layer screening. This new excitonic physics is therefore tied to the basic physics of a gated bilayer geometry, and goes beyond the example we have presented. We conclude that the electric field induced excitonic physics of the TMDC bilayers will exhibit two distinct types of excitonic behaviour: (i) in-plane excitons will become, following the universal trend, less strongly bound as the field closes the gap but, (ii), inter-layer excitons will not follow this universal trend, and the binding energy will be unchanged once the inter-layer screening falls below some critical value. The electric field induced excitonic physics of bilayer TMDCs therefore offers an unexpectedly rich degree of control over the exciton binding energy, a feature that may be of considerable importance in opto-electronic applications.

## Computational Details

### Geometry

The calculations are performed using a super-cell geometry with a large vacuum^21^ of 30 Å (56.7 a.u.) and an in-plane lattice parameter fixed to the bulk experimental value of 3.160 Å^28^. The details of the AA’ stacked configuration that we calculate may be found in ref. 26. We fix the interlayer separation to the same value at all fields; the gap reduction of the *GW* calculations (but not the DFT calculations) is then found to agree closely with experiment. We note that relaxing this constraint has, in one work^25^, been reported to dramatically impact the gap reduction with field, however in such a way that brings the results into sharp disagreement with experiment^5^.

### Electronic structure calculations

The ground-state density functional theory (DFT) calculations are performed using the Perdew-Burke-Ernzerhof (PBE)^29^ exchange-correlation functional using the PAW method as implemented in the VASP^30^ code. We have employed the non-self-consistent *GW* method^31^ to determine the many-body gaps, with excitonic properties calculated by solving the Bethe Salpeter equation (BSE)^19^ in the **q** → 0 limit. Optical spectra was calculated using an energy cutoff of 400 eV, a Gaussian smearing of 0.02 eV, 200 unoccupied bands, and a force tolerance of 0.01 eV/Å. We have included 50 **G**-vectors to account for local field effects. Spin-orbit interaction is included in all calculations. The ELK code^32^ has been deployed to verify the veracity of the BSE results.

### **k**-point convergence

**Figure 6:**
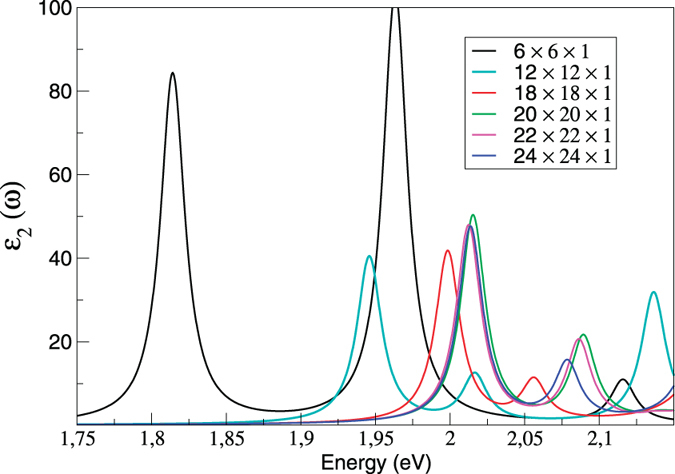
The imaginary part of the in-plane dielectric function of bilayer MoS_2_. Results are calculated with different number of **k**-points.

In order to obtain reliable binding energies it is of vital importance to fully converge the optical spectra. As in a previous work^21^, we find that excitonic binding energies are very sensitive to the number of **k**-points. As is clear from Fig. 6 a minimum grid of 18 × 18 × 1 is required to obtain reliable binding energies (to an accuracy of 0.01 eV), which are then fully converged at a grid size of 24 × 24 × 1. Hence all the results presented in previous sections are calculated using a **k**-point grid of 24 × 24 × 1.

### Electric field

The electric field is included as a scalar potential in the Kohn-Sham Hamiltonian. This potential has a saw-tooth shape, a fact that follows from the imposition of periodic boundary conditions, and gradient of this scalar potential, which is constant across the unit-cell, is then the electric field. Due to this extra term in the Hamiltonian, the Kohn-Sham eigenvalues and eigenvectors are modified. These quantities then lead to an implicit presence of the electric-field in the single particle Green’s function G_0_ and the RPA dielectric function (i.e. the screening), *ε*^−1^, and hence the screened Coulomb potential *W*_0_ = *ε*^−1^*v*.

## Additional Information

**How to cite this article**: Azhikodan, D. *et al.* An anomalous interlayer exciton in MoS_2_. *Sci. Rep.* **6**, 37075; doi: 10.1038/srep37075 (2016).

**Publisher's note:** Springer Nature remains neutral with regard to jurisdictional claims in published maps and institutional affiliations.
